# A complete, evidence-based review on novichok poisoning based on epidemiological aspects and clinical management

**DOI:** 10.3389/ftox.2022.1004705

**Published:** 2023-01-25

**Authors:** Amirhosein Charejoo, Masoud Arabfard, Amir Jafari, Yazdan Hasani Nourian

**Affiliations:** Chemical Injuries Research Center, Systems Biology and Poisonings Institute, Baqiyatallah University of Medical Sciences, Tehran, Iran

**Keywords:** NOVICHOK, toxicity, poisoning, prognosis, clinical manifestations

## Abstract

**Background:** The whole world has learned about the existence of a highly toxic neuro-paralytic substance called Novichok. A wide range of neuro-paralytic toxins were used during the wars of decades ago, which also had harmful and irreversible effects. Fortunately, the establishment of conventions prohibiting the use of these weapons prevented the adverse clinical consequences of these compounds. What we did in the present study was to evaluate the clinical features of Novichok, how to manage exposure to it, and to evaluate the prognostic aspects associated with this poisoning agent.

**Methods:** The manuscript especial databases including Medline, Web of knowledge, Google scholar, and Scopus were deeply searched by the two blinded investigators for all eligible studies based on the considered keywords. Initially 98 articles were initially collected by database searching that considering eligibility criteria, 83 articles were finally eligible for the final assessment. There is a lack of clinical trials and case-cohort studies on general population about treatment and side effects when it comes to human nerve agents and most of the data in our search is based on animal studies.

**Results:** In evaluating various clinical, auto physiological and prognostic aspects of exposure to these substances, special attention was necessary to the following points. First, Novichok agents are considered more potent than other toxic agents. Pathophysiologically, these agents irreversibly bind acetylcholinesterase and produce a rapid cholinergic toxidrome which is responsible for the clinical manifestations as well as the potential dangerous and life threatening side effects caused by these agents. Uniquely, these agents are thought to also target every neuron in the central and peripheral nervous system. As a managerial and therapeutic approach, early and timely treatment of its related complication along with prevents massive exposure and decontamination in addition to rapid resuscitation can prohibit debilitating neuropathy and death due to facing it.

**Conclusion:** The present review highlights the importance of recognizing the potential acute toxic effects of Novichok agents, diagnostic and therapeutic approaches (life-saving antidotal therapy) to complications and ultimately the application of guidelines to improve the prognosis of exposure to these agents for both victims and medical community.

## Introduction

**FIGURE 1:**
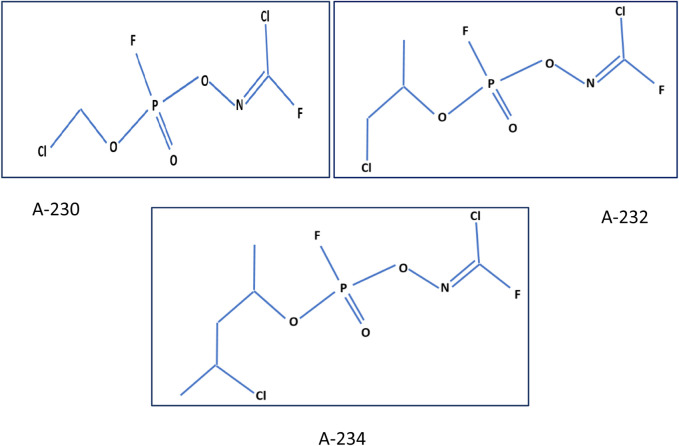
Novichok structures (A-230, A-232, A-234) (Kloske and Witkiewicz, 2019).

Nerve agents are categorized as the G agents [GA (Tabun), GB (Sarin), GD (Soman), GF (Cyclosarin)], the V agents (VX, methylphosphonothioic acid) and novel agents (A-series compounds) (Hulse et al., 2019). Which are less known, and called “Novichok”; an equivalent word for “newcomer” in Russian (Hulse et al., 2019; Holmgaard and Nielsen, 2009). The LD50 (dose required to kill 50% of exposed patients) of Novichok agents is approximately 22 g/kg, which is approximately equal to the lethality of 2-(dimethylamino) ethyl dimethyl phosphoramidofluoride (VG) (Johnson, 2016). Since Novichoks have fewer conformers than VX (6561 for VX, 486 for A-234, and 54 for A-230), these novel agents can bind the acetylcholinesterase enzyme more quickly and lead to its faster transformation (Loyola, 2019). Because of its rapid action in inactivating the enzyme and nearly similar fatality, Novichoks can be considered even more hazardous than VX (Loyola, 2019). The central part of the information we know about these agents is from Dr. Vil Mirzayanov, a Russian scientist (Chai et al., 2018). Novichok compounds’ molecular structure was originally described in 2007.; A-230, A-232, and A-234 (Figure 1.), that structured on alkylphosphorofluoridate scaffolds with carbonimidic substituents (Ellison, 2007). Dr. Mirzayanov also contributed A-242 and A-262, which are based on alkyl phosphonamidofluoridate and alkyl phosphoramidofluoridate scaffolds with guanidyl substituents. These newly added compounds have more nitrogen atoms than A-230, A-232, and A-234, hence they are not covered by the joint proposal (Mirzayanov, 2008). According to the findings of a 2019 study, A-232 is more volatile and less stable in moisturizers than A-230, and has the same toxicity as VR. However, there is insufficient evidence to show A-234s toxicity. A-230 and A-242 have an alkyl group directly linked to the phosphorus atom, but the other three structures (A-262, A234, and A-232) have an alkyl group coupled to the phosphorus atom *via* an oxygen atom, according to this study (Costanzi and Koblentz, 2019). Recently, a group of Iranian scientists in Semnan University have synthesized analogs of A-242 for analytical chemical purposes (Hosseini et al., 2016).

The initial step of the synthesis reaction is the cyclic oxime formation and getting the phosphorus atom five-bound. Then the ring with chlorine transfer opens and Novichok emerges (Kloske and Witkiewicz, 2019). According to Hoenig, there are almost 50 chemical precursors to *Novichoks* (Hoenig, 2007). The techniques of Novichoks analysis are not well described like other nerve agents, and based on their mechanism and structure, these methods are mainly similar to G and V agents (Witkiewicz et al., 2018). These agents are supposed to present an organophosphorus core with P=O, P=S, or P=Se bonds, featuring fluorine as a leaving group and other organic classes like phosgene oxime and analogs for boosting toxicity (Bajgar, 2012; Chai et al., 2018). In the paper “Salisbury Novichok attack: 5 minutes with Christine Blanshard”, we read about a doctor who talks about his experiences regarding the management of the victims of the Salisbury assassination in 2018. He described that event as the longest-running major incident in the NHS, lasting about 72 days (Rimmer, 2019).

In the presence of chemical agents, therapeutic and supportive measures are critical. In fact, in the face of new agents like Novichok, these procedures are more general due to a lack of expertise (Wu and O'Sullivan, 2019). These precautions involve three steps in the case of a Novichok: decontamination, resuscitation, and specific treatment (Kuca and Nepovimova, 2019). Novichok treatments are remarkably similar to those for other organophosphates. In fact, oximes and atropine are the most significant antidotes and treatments in this case as well (Hulse et al., 2019). In addition, new therapeutic options to handle Novichok have been investigated in numerous researches, which we have described in depth in this study.

The consequences of pathological toxicity, epidemiology, toxicity mechanisms, and treatment approaches of neurological factors, particularly the Novichok factor, were addressed in this review.

## Methodology

**FIGURE 2:**
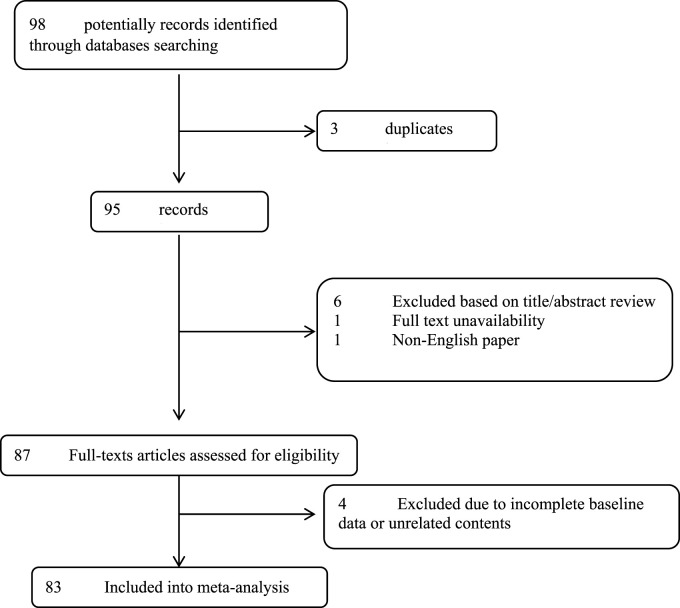
The flowchart of screening the eligible studies.

Firstly, the main study questions were suggested based on the authors purposes as “What is the epidemiological aspects of different nerve poisoning agents?“, “what are the main clinical manifestations of such poisoning?“, and “Which of the ways for decontamination due to such poisoning?” In the next step, the manuscript databases including Medline, Web of Science, Google scholar, and Scopus were deeply searched by the two blinded investigators for all eligible studies based on the considered keywords including “Novichok”, “chemical warfare”, “poisoning”, “epidemiology”, “mechanism”, and “nerve”. The inclusion criteria were considered to retrieve the studies: 1) the studies finally assessed different aspects of Novichok poisoning clinical manifestations, management, and its clinical prognosis and published between 1989 and 2020, 2) The studies were restricted to English language, 3) the studies with unclear or irreproducible results were all excluded, 4) lack of access to the manuscripts full texts was also considered as the inclusion criteria unless the abstracts had enough data for our analysis, 5) case reports and review papers were all excluded. As shown in the flow diagram of the study selection (Figure 2), 98 articles were initially collected by database searching. After removing three articles due to evidences of duplication, 95 records were primarily under-screened. Based on the titles and abstracts, 8 records were excluded and the remaining 87 citations were assessed for further eligibility. Of those, four were also excluded due to incompleteness of the data and contents. In final, 83 articles were eligible for the final assessment.

### Epidemiology of different nerve agents

Nerve-destructive agents are one of the most well-known terrorist agents. There are only handful chemical agents capable of killing huge groups of humans. Like methyl isocyanate release that leads to 2,500 severe and 200,000 injuries in the industrial accident in 1984, or the Tokyo subway Sarin (like attenuated *Novichok*) release in 1995 with 12 deaths and 5,500 injuries (Broughton, 2005; Varma and Varma, 2005). Poisoning is a global problem that consumes a lot of healthcare resources and causes a lot of deaths. The developing world bears the burden of serious poisoning. However, in the developed world, the morbidity and mortality induced by poisoning are also a significant public health concern (Bertolote et al., 2006).

Annually, more than 300,000 deaths are reported due to pesticides around the world (Bertolote et al., 2006). Some nerve agents like Novichok, sarin, VX are more potent and dangerous than others and can be used as potentially fatal chemical agents (Loyola, 2019).

During World War II, scientists in Germany discovered and manufactured three nerve agents, classified as GA (tabun), GB (sarin), and GD (soman). In 1952, VX and GF were manufactured by chemists in the United Kingdom and US (Brennan et al., 1999; Nepovimova and Kuca, 2018). These agents were chosen as chemical warfare agents because they had greater effects on the central nervous system with higher lethality as compared to many of the organophosphates and carbamates used for insect control (Nepovimova and Kuca, 2018).

After the Cold War, with both the US and USSR Soviet Union developing large stores of these toxic agents, organophosphate production boosted (Mirzayanov, 2008). In the 1980s, Iraq could manufacture these agents extensively and used Sarin (GB) and Tabun (GA) (Balali-Mood et al., 2016) against its own Kurdish people and also Iranian people in a prolonged border war (Sidell et al., 1997). The assassination of Kim Jong Nam by a VX toxin at Kuala Lumpur Airport in 2017 was one of the most well-known uses of these weapons. Nerve agents also were confirmed to be used during the Syrian civil war in 2013, and lead to over 800 civilian casualties (Dolgin, 2013).

Nevertheless, Novichok agents have never been used on battlefields, but they have attracted attention with recent assassinations in the United Kingdom. The First documented toxicity occurred in 2018, when Sergei Skripal and his daughter were poisoned with A-234 in Salisbury. During the investigation into this event, two police officers were also poisoned. One month later, toxicity was also reported in Amesbury, in which a man and his wife were poisoned by a bottle of perfume (Kloske and Witkiewicz, 2019).

The events in Salisbury made the United States, the Netherlands, and Canada in 2018, and then Russia in 2019, to propose the Chemical Weapons Convention (CWC) amend their schedules regarding Novichoks (Costanzi and Koblentz, 2019). These or comparable agents can now be produced in a number of countries. Terrorist groups can also use them. The Aum Shinrikyo utilized home sarin in two different attacks, one of which killed seven people in Matsumoto in 1984 (Ivarsson et al., 1992).

### Mechanism of action, signs, and symptoms

The suppression of carboxyl ester hydrolases, particularly acetylcholinesterase (AChE), is the major mode of action of organophosphate compound. Acetylcholine (ACh) is a neurotransmitter that is degraded by AChE into choline and acetic acid. Organophosphates phosphorylate the serine hydroxyl group at AChE’s active site, rendering it inactive. Phosphorylation is followed by the loss of an organophosphate leaving group over time, resulting in the irreversible connection with AChE, a process known as aging. When AChE is inhibited, ACh remains at its postsynaptic receptor sites, leading to immoderate cholinergic stimulation. There are many cholinergic receptors on the tear, sweat, and bronchial secretion glands, and also on the sinoatrial and atrioventricular nodes of the heart in the parasympathetic nervous system (Hulse et al., 2019).

Excessive activation of these receptors stimulates the cholinergic system, resulting in excessive secretion of bodily fluids such as saliva, tears, and urine. Increased cholinergic system activation can also cause bradycardia, bronchospasm, and miosis; which called as the “DUMBBELLS” syndrome (Stapczynski et al., 2019). Also, the nicotinic receptor or the neuromuscular junction is another site where cholinergic transmission is affected. Cholinergic stimulation leads to the progression of deep muscular weakness leading to total paralysis in a dose-dependent fashion and irreversible neuropathy. At the initial stages of exposure, paradoxical hypertension and tachycardia may be seen. However, they decrease as the bradycardia from muscarinic receptors predominates in the clinical manifestation. The last important location which can be affected by cholinergic stimulation is the nervous system. Some organophosphates can easily breach the blood-brain barrier, causing a variety of symptoms such as loss of consciousness, difficulty to breathe, and seizures when used as a nerve agent. These signs and symptoms can be used as a method to detect which nerve agent was used (Hulse et al., 2019).

The risk to healthcare providers from an organophosphate (OP) poisoned patient seems low, especially if appropriate decontamination is performed and staff wears proper personal protective equipment (PPE), including full or half-face masks with air filters, chemical resistant inner and outer clothing, gloves and boots. Medical staff must ensure that large stockpiles of atropine and oximes are available and use them immediately to reduce the burden of probable hazardous incidents (Hulse et al., 2019).

### General management

#### First step: Decontamination

The importance of decontamination is due to both avoiding the ongoing poisoning of the victim and also protecting healthcare staff. To initiate this step, all staff should wear suitable personal protective equipment (PPE). The decontamination type generally depends on the features of the toxic substance and its exposure route if known. Life-threatening injuries such as airway defects must be managed at the beginning to save the life of the patient (Ivarsson et al., 1992; Holmgaard and Nielsen, 2009).

Disrobing the victim (removal of clothing) will provide at least 80% of the decontamination because clothing fibers have the ability to trap and hold liquid nerve agents and their vapors (Balali-Mood et al., 2016). For decontamination of liquid agents, especially lipophilic agents, using clinical paper towels (blue rolls), or some adsorbent powders named Fuller’s Earth and then rinsing them off with warm soapy water, may be required. All clothing and accessories of the patient should be bagged and placed in a well-ventilated outdoor area. This can prevent off-gassing agents from spreading (Table1) (Holmgaard and Nielsen, 2009; Balali-Mood et al., 2016).

**TABLE 1:** Decontamination rules for different forms of nerve agents.

Form of poison	Management
Gas	Moving to a ventilated area
Liquid	Disrobing, dry and wet decontamination (applying adsorbents)
Solid	Disrobing, Applying a face mask to the victim, wet decontamination
Vapor	Disrobing

#### Second step: Resuscitation

The priority in managing any poisoning is performing correct resuscitation, which begins with checking the airway and breathing. Subsequently, other steps such as using antidotes, decontamination, or improved elimination techniques are used to treat poisoned patients. One of the most vital tasks for patients is preparing for appropriate supportive care and observation. In summary, following exposure to CBRN (chemical, biological, radiological, and nuclear) weapons, immediate toxidrome recognition, and using proper antidotes, and suitable decontamination are effective in-hospital care and the main principles of the management of chemical casualties (Holmgaard and Nielsen, 2009).

Treatment of cardiac arrest in poisoned patients requires performing the Advanced Cardiac Life Support (ACLS) guidelines with helpful interventions for toxin-induced cardiac arrest (Stapczynski et al., 2019). Therapeutic management of cardiac arrest has two main goals: initial management of shock and organ failure, and maximum cerebral protection. Using extracorporeal cardiac and respiratory assist devices until multi organ toxicity resolves can rescue patients (Mongardon et al., 2011). The initial critical measures include airway, breathing, and circulation stabilization. Unsuitable ventilation might result from patent airway or reduced respiratory drive, and to avoid it, utilizing mechanical airway and assisted ventilation is essential (Stapczynski et al., 2019). Like most resuscitation circumstances, the first-line choice to treat the hypotension is IV crystalloid bolus (10–20 mL/kg). If the patient is not fluid depleted, then excess fluid administration must be prevented. Although the appropriate volume is replaced, a special antidote may resolve the persisting hypotension. Otherwise, an inotropic agent must be administered cautiously. The assessment of toxicodynamic properties should be considered in choosing an adequate inotrope substance (Committee, 2002). For patients suffering from cardiovascular failure, using extracorporeal membrane oxygenation is essential. Making the airway, breathing, and circulation stable gives the ability to assess other vital signs such as temperature, conscious state, and blood glucose concentration (Mongardon et al., 2011). The next step after the initial general measurements is to use the proper antidote, which is specific for each toxin. However, a few cures are used before cardiopulmonary stabilization, for instance, Atropine for organophosphate toxicity Table2 (Tareg, 2001; Committee, 2002; Mongardon et al., 2011).

**TABLE 2:** Triage for nerve agent casualties (Tareg, 2001).

Classification	Effects	Clinical manifestation
Immediate		
1	Unconscious, talking but not walking, or moderate to severe effects in ≥2 body systems	seizure, severe respiratory failure or apnea, recent cardiac arrest
Delayed		
2	Recovering from agent exposure or antidote	Decreased secretions, improving respiration
Minimal		
3	Walking and talking	Miosis, rhinorrhea, mild to moderate dyspnea
Expectant		
4	Unconscious	Prolonged Cardiac/respiratory arrest

### Third step: Specific treatment

#### Prophylaxis

To prevent further poisoning, delay anticipated toxicities, or reduce their severity; there are some methods used, mainly in the military, as prophylactic strategies. One of the effective ways is to use a dermal tropical patch which contains a mixture of polytetrafluoroethylene and perfluoroalkylpolyether in equal proportions (Skin Exposure Reduction Paste against Chemical Warfare Agents, SERPACWA). Non-etheless, these measurements do not eliminate the necessity of observing PPE and other protective actions. It is recommended to clean the skin with a dry towel before using the mixture (Levitin et al., 2003). The study analyzed the effects of SERPACWA, RDSL (Active Skin Disinfection Lotion) and M291 Skin Disinfection Kit and a mixture of .5% bleach and 1% soapy water on VX-poisoned guinea pigs. The results showed that RSDL had the best protection, while the M291 SDK had the least protection, and finally, .5% bleach, 1% soapy water, and SERPACWA had less protective effects than RSDL (Braue et al., 2011).

SERPACWA is not used in the US army anymore, and other topical dermal protective agents, like IB1 (Israel), and AG7 (United Kingdom) have manifested more efficacy (Timperley et al., 2019).

In many studies, the prophylactic agents are the same as the pretreatment ones. In a survey by Myhrer and Aas in 2016, the use of HI-6, levetiracetam, procyclidine, and HI-6, scopolamine, physostigmine because of their good anti-convulsing effects despite their probable behavioral side effects, is recommended as the prevention of anticipated poisoning (Myhrer and Aas, 2016).

### Pretreatment

**FIGURE 3:**
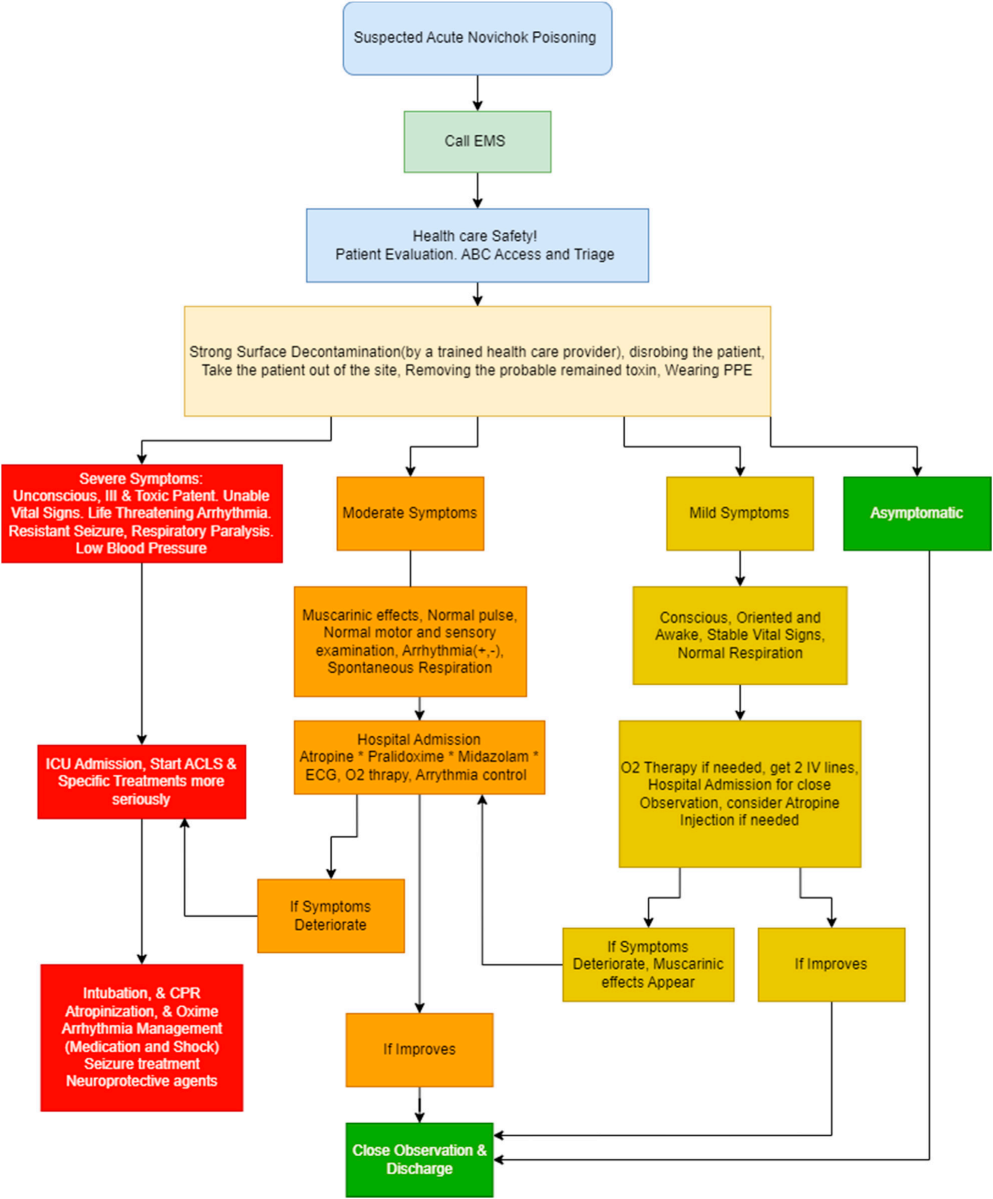
The clinical management approach in exposing Novichok (nerve agent) poisoning.

Pretreatment is different from prophylaxis, and an appropriate pretreatment can enhance the results of following correct treatment (Timperley et al., 2019). The only FDA-approved medication for pretreatment of OP poisoning is Pyridostigmine bromide (30 mg every eight orally), a carbamoylate with the ability to avoid the OP inhibitor binding (Harris et al., 1989; Tareg, 2001). Two hours before the first dose, proper care against lethality should be started and continued at least till the third dose. Pretreatment with Pyridostigmine must be ceased before the nerve agent poisoning symptoms (Figure 3) occur (van Helden et al., 2011). In some studies, Rivastigmine revealed high inter-patient variability because of its complex pharmacological profile (Lavon et al., 2015; Park et al., 2018). Furthermore, Galantamine, based on some preclinical studies, seems to be potent and effective with low toxicity (Indu et al., 2016). Other animal investigations have demonstrated the efficacy of drugs like as Galantamine, Physostigmine, Trihexyphenidyl, Benactyzine, and Procyclidine; however, in anticonvulsant doses against Soman, they all resulted in behavioral impairments (Myhrer and Aas, 2016).

In 2019, Myers did a study to evaluate the efficacy of HuBChE (Human Butyrylcholinesterase) in the reduction or prevention of OP poisoning. This animal study showed successful results and HuBChE remains a good bio scavenger for both prophylaxis and treatment of OP poisoning Nevertheless, this method is not affordable on large scales like battlefields (Myers, 2019).

Another productive method being used as pretreatment is stoichiometric scavengers, which ensure suitable protection against high doses of Soman. But no important side effects were reported in animals (Myhrer and Aas, 2016).

Besides the importance of pretreatment, we, as physicians facing novichok poisoned patients, have to start the lifesaving atropine as an initial dose of 5–10 mg intravenous/intraosseous followed by additional doses every 5 min until atropinisation happens (reversing of 3b: bradycardia, bronchospasm, bronchorrhea). It is important to start oxime and anticonvulsant drugs (if needed) simultaneously.

#### Atropine

Suppression of cholinergic excess at all three sites is the primary strategy for treating the toxicity of nerve agents. Atropine suppresses only the muscarinic effects, while Pralidoxime counteracts the nicotinic effects (Eddleston et al., 2008).

Although the influential role of atropine as the first-line pharmaceutical management of OP poisoning has been proved several years ago, there are some limitations that made scientists try more productive alternatives. The most critical barrier to atropine is its inability to cross the blood-brain barrier (BBB) firmly to control CNS deficits, for instance, convulsions due to nerve agent poisoning (Chai et al., 2018; NATO, 2018).

Intravenously administration of atropine should be considered in poisoned people suffering from hypersalivation, bradycardia, or bronchial secretions. Atropine titration should be performed to clear the excess secretions. The recommended amount to initiate Atropine is 2–5 mg for mild, but 5–10 mg intravenous IV)/intraosseous (IO) for severely poisoned victims, which is in the protocol presented by the United Kingdom military and North Atlantic Treaty Organization (NATO) (NATO, 2018). Indeed, the recommended dose is four–4.2 mg as an initial dose in severe nerve agent cases, according to the Public Health England guidance. It recommends using a 1–3 mg bolus, then doubling the dose every 5 min until atropinization happens, and finally atropine infusion (Eddleston et al., 2008). A study by Timperley in 2019, suggested the initial amount for severe cases was 6 mg IV or intramuscular (IM), and then 2 mg IV or IM, every 5–10 min (Timperley et al., 2019). It should be titrated every 5 min until atropinization (defined as systolic BP > 80 mm Hg, heart rate HR > 80 beats/min, and drying of pulmonary secretions) occurs and also bradycardia, bronchospasm, and bronchorrhea improve (Holmgaard and Nielsen, 2009). Infusion after intravenous administration is preferred. Since, toxicity from large doses of atropine is inevitable, close observation of the patient and checking the vital signs is essential. ECG monitoring, if possible, should be done (Lavon et al., 2015), (Perera et al., 2008). In an observational study by Perera et al., in 2008, two types of common atropinization in patients with acute organophosphorus and carbamate poisoning were reviewed. The major aim of their study was to assess the outcomes of mortality and ventilation. Victims in the titrated dose cohort showed clinical signs compatible with more excellent toxicity, probably because of more toxic OP agent ingestion. They finally concluded that high-dose atropine regimens do not enhance the outcomes of patients, but result in more frequent atropine toxicity. Thus, antidote titration is recommended in the management of all patients. (Perera et al., 2008). The effects of atropine last around 3–5 h after one or two injections of 2 mg, and 12–24 h after over atropinization (Timperley et al., 2019). It is possible that high dosages of atropine IV, up to 20 mg, will be required. These doses are typically required when ingestion is by swallowing. However, they are rarely required when exposure is by other means. Because of the low quantities, substantial dosages of the antidote were not required in the Tokyo subway accident (Balalimoud and Balali, 2008). In massive exposures, with limited resources, the use of ophthalmic atropine drops, and veterinary atropine are recommended.

Atropine given prior to oxygen did not increase the fatality rate in OP poisoning sufferers. As a result, if oxygen is not available, it is not suggested to stop delivering atropine to critical patients (OPCW, 2018).

In addition, many studies have focused on the effect of using MgSO4 on the whole management and consequences of nerve agents poisoned patients. Sulfate Magnesium by inhibiting calcium channels counteracts presynaptic acetylcholine release. A randomized control study in 2017, concluded that the infusion of 4–6 g of 20% MgSO4 solution in 24 h can reduce atropine requirements, intubation quantities, and ICU days in toxicities with OP (Eddleston and Chowdhury, 2016; Vijayakumar et al., 2017; Brvar et al., 2018; Jamshidi et al., 2018).

#### Alternatives for atropine

Glycopyrrolate is one of the medications which have attracted attentions as an alternative to Atropine. Many studies have been done to assess the effects of this drug administered alone, or in combination with other agents. In a study, the combination of atropine and Glycopyrrolate was observed and it was presumed to show the proper potency in controlling OP poisoning symptoms and lessening the central neural toxicity because its effects are limited to the PNS. This study observed 53 patients from 2003 to 2006 at Tygerberg Academic Hospital (TAH). Though two patients treated for OPP expired, the mortality rate was lower than that previously reported there (Arendse and Irusen, 2009). Contrarily, a retrospective study revealed that using Atropine alone causes fewer complications than combinational therapy with Glycopyrrolate (Bhandarkar, 2014). Moreover, few studies recommended that using atropine alone results in lower OP-induced mortality (King and Aaron, 2015). Some studies have suggested antihistamine agents like diphenhydramine and Promethazine have acceptable antimuscarinic effects (Mohammad et al., 2012; Ojha et al., 2014; Nurulain et al., 2015). In cases of hypersensitivity, atropine can be substituted with Scopolamine or Glycopyrrolate. A recommended dose for Glycopyrrolate is 1 mg IV every 10–15 min till anti-muscarinic effects happen. If IV access is unavailable, IM administration can be considered every 30–40 min (Martellini and Trapp, 2020).

Scopolamine is another medication which has shown efficacy in many studies. Cornelissen et al., in 2020, declared that Scopolamine and Atropine manifested similar bioavailability in their study, but Atropine provided lower CNS levels. This effect is associated with an improved anticonvulsant effect of Scopolamine (Cornelissen et al., 2020). Scopolamine should be given initially at 2–0.6 mg IM or IV. In patients with isolated pulmonary symptoms, choosing Ipratropium Bromide may be useful to clear secretions (Bird et al., 2003). Based on the latest studies by Katz et al., in 2018, Glycopyrrolate can be considered in patients with recurrent symptoms after first atropinization, but those patients may suffer from central anticholinergic delirium or agitation (Katz et al., 2018).

The IM auto-injection of Atropine (2 mg) should be administered once in the case of mild symptoms. After 10–15 min, if severe symptoms occur, two additional injections should be considered, otherwise extra doses are not required. In really severe cases, three injections can be used into the patient’s mid-lateral outer thigh rapidly (Katz et al., 2018). The key point is that all of these alternatives to atropine should be used carefully and in the case of hypersensitivity to atropine because there is not enough evidence to show they are doing better than atropine.

#### Pralidoxime

Pralidoxime (PAM) was the first synthetized oxime which counteracts nicotinic effects. It is commonly used in the US, France, and the United Kingdom, while other oximes are more utilized in other European countries (Balali-Mood et al., 2016). Pralidoxime can reactivate acetylcholinesterase at the neuromuscular junction only if the aging process has not happened. The rate of aging in different agents is different; VX takes 24 h, while this time is just 5–8 min for Soman, and it creates an irreversible bond (Hulse et al., 2019). The only FDA-approved oxime derivative currently available is 2-pralidoxime chloride (2-PAM), which has enough potency. It has also been prepared as an IM auto-injector type containing 600 mg/2 mL. However, the preferred route in hospitals is IV administration (Stapczynski et al., 2019). The suggested loading dosage of Pralidoxime is a slow IV administration of 1–2 g in 100 mL of N/S serum over 20–30 min, 600 mg can be given intramuscularly for up to three doses. However, half of this amount might be enough if the volume of the event is large and the number of casualties is high (Chai et al., 2018). The DuoDote kits for auto injection contain 600 mg of pralidoxime chloride and 2.1 mg of Atropine (Newmark, 2019). Another FDA approved auto injector is ATNAA, which contains a mixture of 2-PAM, 600 mg/2 mL and atropine, 2.1 mg/0.7 mL, in two different chambers (Traynor, 2014). Studies suggest strongly using three DuoDotes for any victims with serious respiratory or CNS manifestations at the moment (Newmark, 2019).

Previous studies recommended continuing the administration of oximes for at least 12 h (>24–48 h until recovery of the patient) after reactivation and patient recovery (Timperley et al., 2019). All patients who received Pralidoxime must be admitted to an intensive care unit to get either a continuous infusion of 500 mg/h Pralidoxime, or intermittent bolus dosages of 1–2 g every 6 h (Eddleston et al., 2008).

If oximes weakly penetrate the blood brain barrier, and are not able to reverse the muscarinic effects such as hypersecretions, atropine should be simultaneously administrated. Although Pralidoxime is the first known oxime which has shown efficacy in OP toxicity, there are some limitations that make scientists present new agents. Based on several studies, the presently-used oximes can only about 4%–10% of the plasma level penetrate the blood brain barrier, and this amount is not enough to protect the brain against the toxin (Lorke et al., 2008).

#### Other oximes

Methoxime, or MMB-4, is a bis-pyridinium oxime that has shown improved pharmaceutical properties and therapeutic range compared to 2-PAM and obidoxime (Lundy et al., 2011; Machamer et al., 2020). In addition, Asoxime Chloride (HI-6) is a substantially broad-spectrum AChE reactivator, and a double-positive charged bispyridinium compound, commonly used in Canada, Sweden, and the Czech Republic. It has shown acceptable effects in treating Sarin, VX, Soman and cyclosarin (Kassa et al., 2007; Cuya et al., 2018; Whitmore et al., 2018; Kranawetvogl et al., 2020). Timedoxime bromide, or TMB4, is another oxime that is particularly successful in regenerating AChE, although its use is restricted due to liver and CNS side effects (SZ et al., 1985). Moreover, Obidoxime is another oxime which is used particularly in Germany and the Netherlands. It is effective for Tabun poisoning, unlike Asoxime, and also has some anti-muscarinic activities (Soukup et al., 2011). A suggested dose is 250 mg IV bolus, followed by 750 mg over a 24-h period (Thiermann et al., 1997). Recently, a new generation of oximes, called K-oximes, has been developed that enhances AChE regeneration (Petroianu et al., 2007; Gorecki et al., 2019). In a study conducted in 2022, the results showed that commercial oximes have limited efficiency for the reactivation of Novichok-inhibited acetylcholinesterase enzyme (Ser203-A-242), since they are not able to properly approach the adduct Ser203-A-242. Among those oximes, trimedoxime seems to be the most promising (This study used computational techniques such as molecular docking, molecular dynamics and MM-PBSA interaction energy calculations.) (Santos et al., 2022a). Also, another study showed trimedoxime as the most promising commercial oxime for the reactivation of AChE inhibited by A-242 (Santos et al., 2022b). Enough human studies have not been conducted about these new oximes, so we, as physicians, must use the first oximes available in our drug stocks with lifesaving atropine in case of novichok poisoning.

### Successful advanced therapies

Recently, many experiments apart from the initial remedies have been conducted to promote the outcomes associated with the existing therapies, which may lack the potency as expected. For example, the results of a randomized controlled study published in 2017 that examined the effects of red blood cell (RBC) transfusion on increased cholinesterase activity after OP poisoning showed that giving fresh or longer-storage RBC can shorten the time spent on atropine and pralidoxime treatment (Bao et al., 2017).

Lastly, several studies have been conducted to assess the treatment without oximes or with modified forms. In general, there have been two strategies to inhibit OP activity, and both should be considered if a satisfying result is desired: first, lowering Ach concentrations in the synapse, and second, restricting ACh activation of muscarinic and nicotinic receptors (Masson, 2011; Timperley et al., 2019). Recent research has shown that site-directed mutagenesis of AChE makes aldoximes able to increase reactivation of OP-enzyme conjugates, whereas the rates of dealkylation (aging) decrease (Kovarik and Hrvat, 2020).

Currently, there is positive evidence for using scavengers as alternate objects for nerve agents. Bioscavengers are exogenous enzymes that have ability to neutralize the OP in the blood stream (Kobrlova et al., 2019). They are believed to reduce nerve agent binding to cholinesterase enzymes. The most noticeable advantage is that scavengers will provide a longer duration of effect in treating victims (Martellini and Trapp, 2020). Based on a study by Kovarik and Macek in 2020, coordinated stereoselective preference for the OP enantiomer for inactivation and reactivation is a benefit of cholinesterase as a bioscavenger (Kovarik and Hrvat, 2020).

Kranawetvogl et al., in 2020, introduced a new, simple, and rugged HPLC-DAD (High-Performance Liquid Chromatography with Diode-Array Detection) technique, which is functional for the single analysis of Obidoxime, 2-PAM, and HI-6 in human plasma by using 4-pyridinealdoxime as the internal standard and sensitive DAD detection. Their study focused on fulfilling a successful approach to improving therapies involving the combined administration of different oximes (Kranawetvogl et al., 2020).

Another novel treatment approach, whose efficacy in severe cases has been demonstrated, includes Intravenous Lipid Emulsion (ILE), which reduces the agent’s access to active biological sites, and prepares energy for poisoned myocardium (Hoegberg et al., 2016; American College of Medical Toxicology, 2017).

Presently, *in vitro* investigations have showed that acetylmonoethylcholine (AMECh) and acetyldiethylcholine (ADECh), two ACh analogs, can reduce the ACh receptors’ overstimulation (Whitmore et al., 2018). Furthermore, some studies have demonstrated that diuretics like furosemide and mannitol can improve OP excretion and also control OP-induced edema (Jiang et al., 2019).

Recent findings with the approach to *in vitro* assessment of new non-oximes to reactivate the human acetylcholinesterase inhibited by nerve agents have shown that, apart from oximes, special Mannich phenols are able to reactivate the inhibited enzyme very successfully (de Koning et al., 2020). In a study by Wang and Wang in 2014, utilizing nano carriers illustrated the ability to improve drug transport into the CNS, thereby protecting against enzymatic degradation and slow release (Wang and Wang, 2014). Chambers and Meek in 2020 performed a study and declared to invent novel substituted Phenoxyalkyl Pyridinium oximes by adding an extra lipophilic chain to the Pyridinium ring, which shows the potency in proceeding into the brain to reverse the major part of the nerve agent induced AChE inhibition (Chambers and Meek, 2020). Advanced therapies including RBC transfusion, bio scavengers, Intravenous Lipid Emulsion, special phenols, and site-directed mutant AChE are helpful in lowering the duration of healing procedures and could lead to better prognosis. There is a need for more trial and cohort studies for more curative evidence about them.

### Benzodiazepines

OP poisonings can cause seizures and subsequent brain damage if not controlled properly. The selected specific choice to control convulsions due to nerve agents’ toxicity is Benzodiazepines. For many years, Diazepam has been the first choice, but recently, Midazolam has shown more efficiency in being used as the first line treatment. It can be administered both IM and IV (Reddy and Reddy, 2015). However, Diazepam is well known and familiar to all populations and its auto injectors are available, and also, in many places, there are not any drugs except Diazepam. Lorazepam is another great choice that can be utilized effectively. The recommended dose for Midazolam is 2.2 mg/kg. Diazepam should be administered 5–10 mg IM initially (.2–0.5 mg/kg). The suggested dose for starting Lorazepam is 2–4 mg (.1–0.2 mg/kg). The frequency of later injections depends on the general health condition of each patient, the onset of seizure and the drug dosage (Wu et al., 2018; Timperley et al., 2019).

In the case of occurring seizures, refractory types and diaphragmatic paralysis that causes respiratory arrest should be considered. IV administration of benzodiazepines is used to treat seizures. Phenytoin and Fosphenytoin are not proper choices to control seizures. Observation for a 6–8 h period may just be enough for patients with minimal symptoms, like only eye findings (Stapczynski et al., 2019).

### Neuroprotective agents

Some drugs are recommended after many studies to be used for neuroprotection. Anticonvulsants alone may not control the seizure effectively, thus adding other drugs can improve the result. Memantine is a drug with AChE-protecting and NMDA receptor-blocking action that has been shown to have great efficacy in Soman poisoning. Because of its small therapeutic index and narrow therapeutic window, it should be considered in facing Novichok and other nerve agents’ toxicity because of its positive effects (Stojiljković et al., 2019).

NMDA receptor antagonists, like Gacyclidine or Ketamine (Mion et al., 2003; Vijayakumar et al., 2016), have a high level of neuroprotection regarding nerve agents’ toxicity. The combination of Ketamine and Midazolam in Sarin poisoned rats revealed efficiency as a neuroprotective agent (Lewine et al., 2018). Furthermore, some studies confirmed the use of Ketamine for treating seizures caused by nerve agents poisoning (Mion et al., 2003).

Another development in the field of neuroprotection is the introduction of drugs that are beneficial for OP poisoning induced deliriums. Delirium is one of the most common complications in patients admitted to ICU, and OP poisoning can trigger it. A double-blind randomized placebo controlled trial of 56 OP poisoned patients revealed that using 3 g Melatonin every night is efficacious in decreasing delirium (Vijayakumar et al., 2016). When it comes to human incidents, it is better to use these neuroprotective agents, including memantine, ketamine, and melatonin, with caution to lower neuron damage in the presence of acute nerve agent poisoning.

### Supportive care

Intubation and mechanical ventilation will finally be required for most patients suffering from neuromuscular weakness. For OP poisoned patients, designated machines should be used. However, if needed, the circuit and rubber components of the machine must be replaced and the machine washed before being used again because OP materials can penetrate rubber and plastics (Holmgaard and Nielsen, 2009). Ventilators by circuiting with a Y-piece, and doubling the tidal volume in mass casualty events, can be used for two patients with similar ideal body weight (Menes et al., 2017). In limitations of resources, expired medications or those which are not FDA approved can help a lot and provide relative efficacy, but less than new and approved drugs (Martellini and Trapp, 2020).

### Prognosis

The prognosis of nerve agents’ toxicity depends on the amount of exposure in such a way that high amounts can even result in deaths. However, a study by T. Okumura in 1998, which was performed on 472 staff, revealed that most of them experienced no symptoms after facing exposed victims. Nevertheless, around 23% (*n* = 110) suffered from secondary poisoning with eye symptoms (14%), headache (11%), sore throats (8%), dyspnea (5%), and nausea (3%), which was the slightest symptom (Okumura et al., 1998).

In another retrospective study by Saadeh et al., in 1997, 46 adult patients who were poisoned by organophosphate or carbamate and admitted to MICU were observed over a five-year-period. Around 67% of the whole population (*n* = 31) manifested cardiac complications. These clinical features included non-cardiogenic pulmonary edema in 43% (*n* = 20), and cardiac arrhythmias in 24% (*n* = 11). Also, prolonged Q-Tc interval occurred in 67% of the population (*n* = 31), and ST-T changes in 41% of them (*n* = 19). Conduction defects in 4% (*n* = 4), sinus tachycardia in 35% (*n* = 16), and sinus bradycardia in 22% of patients (*n* = 10) happened. Finally, 17% of patients (*n* = 8) got hypertension after exposure (Saadeh et al., 1997). The risk of mortality increases with serious respiratory and cardiac complications. It is reported that victims of the Tokyo subway attack had hematemesis after being poisoned. Kim Jong-nam expired because of asphyxiation due to VX gas exposure in less than 20 min. Cardiac arrest and respiratory failure are the most important causes of death based on previous studies (Cotton, 2018).

In 2017, Acikalina et al. did a retrospective study with the aim of detecting the factors associated with poor prognosis and mortality. They studied 80 OP poisoned patients from 2010 to 2015 who were admitted to ICU. The results showed that of the underlying comorbidities of the victims, prolonged duration of hospitalization, prolongation of respiratory depression necessitating mechanical ventilation support, elevated levels of plasma creatinine, low level of consciousness and low PcHE (Pseudocholinesterase) levels without regeneration in the initial 48 h of admission are substantially related to poor prognosis following OP toxicity (Acikalin et al., 2017).

In another study in 2009 by Kang et al., concerning the prognostic risk factors and the mortality rates of various OP toxicities, it was demonstrated that the first APACHE II score is a beneficial prognostic factor, and also concluded that various OPs lead to different mortality rates (Kang et al., 2009).

What we must know as a physician is that the health condition of patients coming to the emergency department determines the level of prompt treatment (also see Table 3). Initial blood tests and other para-clinical measurements as well as the general appearance have a vital role in the correct admission of patients. Immediate cardiopulmonary resuscitation can prevent subsequent life-threatening events. Furthermore, rapid response and appropriate management based on standard guidelines can rescue poisoned persons.

**TABLE 3:** Dosage of the recommended drugs.

Medication	Route of administration	Dosage	Frequency
Atropine	IM, IV	2–5 mg then 1–2 mg/h, (5–10 mg for severe cases)	Every 5–10 min
Scopolamine	IM, IV, SC	.2–.6 mg then .6 every 6 h	
Glycopyrrolate	IM, IV	1 mg then .5–1 mg/h	Every 15min IV, or every 30min IM
Pralidoxime	IM, IV	1–2 g IV over 15 min or 600 mg IM then .5 g/h	TID, BID
Asoxime (HI-6)	IV	500 mg then 500 mg QID OR BID	Repeat 2 h after onset, then QID- BID
Obidoxime	IV	250 mg then 750 mg QD	Repeat 2 h after onset,
then QID-BID			
Diazepam	IV, IM	5–10 mg then repeat as required	PRN
Midazolam	IV, IM	5–10 mg then repeat as required	PRN
Lorazepam	IV, IM	2–4 mg then repeat as required	PRN
MgSO4	IV	4–6 g of 20% MgSO4 solution in 24 h	
Pyridostigmine bromide	OP	30 mg	Every 8 h

## Conclusion

Although the number of Novichok (nerve agent) poisoning is low, all physicians must know how to perform the immediate recognition and management to save lives of the victims. The main principles of the management include the early diagnosis and initial resuscitation, adequate decontamination, using Atropine, oximes and neuroprotective agents. These steps should be managed simultaneously without wasting time. The necessity of performing an effective decontamination is both for the healthcare personnel’s safety and for rescuing the patients. After making the patients, stable in a safe environment, specific medications must be administered as soon as possible. For instance, Atropine is the first choice of antidotes. An important clinical point in this step is preventing atropine toxicity by its titration, since high doses of this antidote are not effective in outcome improvement. Another vital compound which is used in conjunction with Atropine and anticonvulsants is Pralidoxime. This drug has some peers in different countries that any of them has special advantages and disadvantages. Obidoxime and Asoxime, for example, are major oximes used in Germany and Canada, respectively. Previous popular medicines may have been ineffective due of pharmaceutical hurdles, such as drug penetration into the brain. As a result, additional research has been conducted to overcome these constraints, as well as several studies to present more options to existing treatments. By way of illustration, some advanced therapies like red blood cell transfusion, using nano particles and bioscavengers, Intravenous Lipid Emulsion (ILE), and some novel oximes have shown success in treating OP poisoned patients. The experiences of assassinations in the United Kingdom and the Tokyo subway incident tell us a lot about the best methods of managing different nerve agents’ toxicities, although there is little information about the new agent, Novichok. This review study forms the basis for future investigations into Novichok agents and their toxicity, since the potentially dangerous nature of this substance threatens the lives of future victims.
